# Plasticity in transmission strategies of the malaria parasite, *Plasmodium chabaudi*: environmental and genetic effects

**DOI:** 10.1111/eva.12005

**Published:** 2012-10-10

**Authors:** Angus Cameron, Sarah E Reece, Damien R Drew, Daniel T Haydon, Andrew J Yates

**Affiliations:** 1 Boyd Orr Centre for Population and Ecosystem Health, Institute of Biodiversity, Animal Health and Comparative Medicine, College of Medical, Veterinary and Life Sciences, University of GlasgowGlasgow, UK; 2 Department of Systems and Computational Biology, Albert Einstein College of MedicineBronx, NY, USA; 3 Centre for Immunity, Infection & Evolution, Institutes of Evolution, Immunology and Infection Research, School of Biological Sciences, University of EdinburghEdinburgh, UK; 4 Burnet InstituteMelbourne, Vic., Australia; 5 Department of Microbiology and Immunology, Albert Einstein College of MedicineBronx, NY, USA

**Keywords:** gametocyte, gametocytogenesis, life history trait, parasite ecology, phenotypic plasticity, resource allocation trade-off, transmission strategies

## Abstract

Parasites may alter their behaviour to cope with changes in the within-host environment. In particular, investment in transmission may alter in response to the availability of parasite resources or host immune responses. However, experimental and theoretical studies have drawn conflicting conclusions regarding parasites' optimal (adaptive) responses to deterioration in habitat quality. We analyse data from acute infections with six genotypes of the rodent malaria species *Plasmodium chabaudi* to quantify how investment in transmission (gametocytes) is influenced by the within-host environment. Using a minimum of modelling assumptions, we find that proportional investment in gametocytogenesis increases sharply with host anaemia and also increases at low parasite densities. Further, stronger dependence of investment on parasite density is associated with greater virulence of the parasite genotype. Our study provides a robust quantitative framework for studying parasites' responses to the host environment and whether these responses are adaptive, which is crucial for predicting the short-term and evolutionary impact of transmission-blocking treatments for parasitic diseases.

## Introduction

Parasites, like all sexually reproducing organisms, must optimize their resource allocation with respect to growth, survival and reproduction (Pollitt et al. 2011a; Mideo and Reece 2012). Here, growth and survival within the host are synonymous with replication and reproduction is synonymous with transmission to subsequent hosts (Koella and Antia 1995; Reece et al. 2009). The allocation of resources to in-host survival and between-host transmission is a key determinant of a parasite's fitness and underpins its tolerance to drugs and the virulence and infectiousness of the associated disease (Mideo and Reece 2012). Developing vaccines or treatments for parasitic diseases are major public health goals. Identifying the factors that shape investment in transmission, and how they do so, may contribute to these efforts by suggesting new therapies to interfere with a parasite's life cycle.

Life history theory is an ecological and evolutionary framework traditionally applied to multicellular taxa (Roff 1992; Stearns 1992) and addresses optimal strategies for investment in reproduction. With some assumptions, for example, it predicts an increase in investment with age as chances of reproductive success decline, as is observed in, for example, macaques (Paul et al. 1993), ants (Heinze and Schrempf 2012) and burying beetles (Cotter et al. 2010). However, age is just one component of an organism's state, a multidimensional quantity that includes, for example, its habitat quality, stored energy reserves or its access to resources. These state components and the interactions between them may influence reproductive investment in complex and species-specific ways (McNamara and Houston 1996; Fischer et al. 2009). Nevertheless, elevated investment in reproduction has been interpreted as an optimal response to adverse habitat quality – a ‘terminal investment' strategy for maximising transmission before the host clears the infection or dies (Williams 1966; Clutton-Brock 1984).

**Figure 1:**
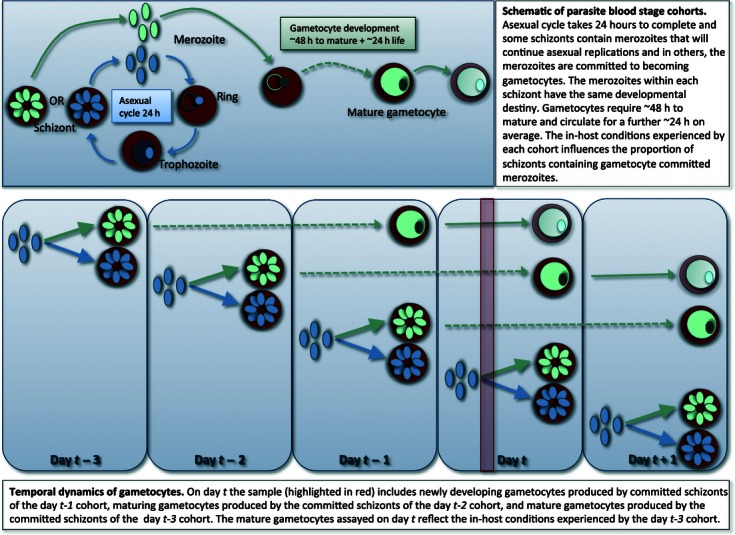
Asexual replication and gametocytogenesis in *Plasmodium chabaudi* infections.

In this study, we focus on *Plasmodium*, the parasite responsible for causing malaria, and specifically on the rodent malaria species *Plasmodium chabaudi*. The parasite's survival within the mammalian host is maintained by cycles of asexual replication within red blood cells (RBC). A small fraction of the parasites produced at each cell cycle differentiate into male and female sexual stages, termed gametocytes, which do not replicate in the host, but are required for transmission (Fig. 1). We refer to this fraction as the *proportional investment* in transmission. (In evolutionary biology, this is also referred to as reproductive effort; in parasitology, the conversion or commitment rate). When taken up in a vector blood meal, gametocytes differentiate into gametes and fertilisation occurs. The requirement of different stages for within-host survival and between-host transmission makes *Plasmodium* a powerful model to study the trade-off between survival and reproduction. Parasites investing heavily in gametocytes early in infections risk curtailing the infectious period owing to insufficient asexual replication to maintain the infection. Conversely, excessive investment in asexual replication reduces the rate of transmission and will also shrink the window for transmission if infections are so virulent that the host dies. Broadly, however, *Plasmodium* invests remarkably little in transmission within the mammalian host, with only a small proportion of merozoites undergoing gametocytogenesis with each round of asexual replication (Taylor and Read 1997; Babiker et al. 2008; Dixon et al. 2008). Several explanations have been proposed for this restraint in its reproductive strategy. It may (i) reduce the virulence experienced by vectors by ensuring only a small number of gametocytes are taken up with any one blood meal (Cohuet et al. 2010); (ii) prevent hosts from developing gametocyte-specific immunity, which would limit transmission (Buckling and Read 2001); or (iii) be an optimal strategy in the context of competition between co-infecting genotypes (Mideo and Day 2008), where most resources are invested in outcompeting conspecifics via asexual replication to ensure future transmission.

*Plasmodium*'s search for an optimal transmission strategy is complicated by its dynamic within-host environment. During the acute phase of infection the host becomes progressively more anaemic as the parasite utilizes RBC to establish itself. The subsequent infection dynamics arise from the interplay between (i) the parasite's continued consumption of host resources, with potentially preferential tropism for certain ages of RBC and/or competition from co-infecting genotypes; (ii) the influx of immature reticulocytes as the result of the host's compensatory increase in erythropoiesis; (iii) innate immune responses, which may be associated with retention of RBC in the spleen and excessive inflammation resulting in the destruction of both infected and healthy RBC; and (iv) adaptive immune responses, likely directed against both shared and distinct antigens expressed by the asexual and sexual stages, and dynamically shifting focus in response to antigenic variation by the parasite (Koella and Antia 1995; Day 2003; Paul et al. 2003; Mideo et al. 2008).

Phenotypic plasticity is a ubiquitous evolutionary solution to the challenges of life in such a changing environment because it gives individual genotypes the ability to express the ‘best’ phenotype in response to its current environmental circumstances. *Plasmodium* indeed appears to exhibit such plasticity; investment in gametocytes varies during infections by genotype and in response to resource availability, drug treatment, and the presence of other parasite genotypes (Trager and Gill 1992; Buckling et al. 1997, 1999a,b; Trager et al. 1999; Reece et al. 2005, 2010; Pollitt et al. 2011b). Is this observed plasticity truly adaptive? To address this question we first need to identify the environmental variables that influence the parasite's propensity to invest in gametocytes, and to what degrees. Understanding how parasites read cues from the host is important as it will identify experimental tests of whether parasite transmission investment strategies are indeed optimal. However, theoretical and experimental studies have yielded conflicting conclusions in this area, particularly regarding the direction of changes in investment in gametocytes in response to changes in the in-host environment. For example, treatment with some drugs has been shown to increase the rate of gametocytogenesis by *P. chabaudi* (Buckling et al. 1997, 1999a) and by *Plasmodium falciparum*, one of the parasites responsible for human malaria (Buckling et al. 1999b), but another study of *P. falciparum* found drug treatment decreased proportional investment in transmission (Reece et al. 2010). Similarly, rates of gametocytogenesis (Pollitt et al. 2011b) and infectivity to mosquitoes, a correlate of gametocytemia, have been found to correlate positively with RBC abundance (Drakeley et al. 1999); gametocytemia was found to be insensitive to changes in RBC availability in avian and murine malaria models (Paul et al. 2000); and field studies of human malarias have shown that gametocytemia correlates positively with anaemia (Price et al. 1999; von Seidlein et al. 2001; Nacher et al. 2002).

Here, to better understand the manner in which environmental factors and parasite density shape transmission strategies, we use statistical models to explore how *P. chabaudi* invests in gametocytes as a function of parasite density and RBC availability in acute single-strain infections of mice.

## Methods

We use a previously published data set (Reece et al. 2008; Pollitt et al. 2011b) from experimental infections of mice with six genetically distinct clones (hereafter referred to as genotypes) of the parasite. Merozoites require 24 h to mature in RBCs and gametocytes require 48 h. Schizogony is synchronized and takes place every 24 h postinfection (PI) (Buckling et al. 1999a; O'Donnell et al. 2011). This synchrony motivated the experimental sampling protocol and invites the use of discrete-time methods such as those used to model the dynamics of total parasite densities (Reece et al. 2008). For *Plasmodium*, the proportion of merozoites that differentiate into gametocytes is a natural readout of its allocation strategy. We predict gametocyte densities as functions of host or parasite factors at earlier time points, accounting for the developmental delay between commitment to gametocytogenesis and observation of mature gametocytes. Using a minimum of modelling assumptions, we test whether and how proportional investment in gametocytes depends on the densities of parasites and mature or immature RBC.

### Experimental design

**Figure 2:**
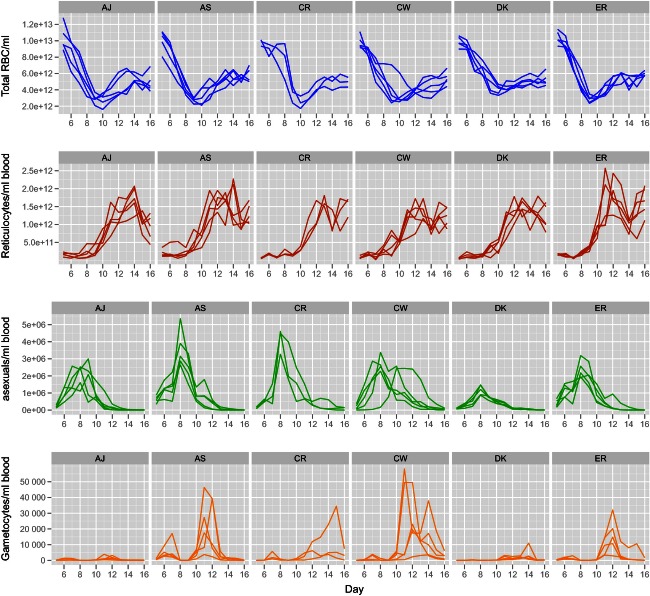
Time courses of red blood cells and parasite densities from day 5 to 16 postinfection with six genotypes of *Plasmodium chabaudi* (AJ, AS, CR, CW, DK, ER). Longitudinal data, five mice per genotype (see Methods).

All experiments were carried out at the University of Edinburgh, UK. *Plasmodium chabaudi* genotypes from the WHO Registry of Standard Malaria Parasites were used. Infections described here were originally set-up to examine sex ratios in single and mixed infections as described by Reece et al. (2008). Here, we consider six single genotype infection groups (genotypes AJ, AS, ER, CR, CW and DK) for analysis. In each treatment group, five mice were inoculated with 1 × 10^6^ parasites. Data are shown in Fig. 2.

Mice used were 6–8-week-old MF1 males (in-house supplier, University of Edinburgh). All mice had blood sampled daily during the acute phase of infection; from day 5 PI until day 16 PI. Sampling took place in the morning to ensure circulating parasites were in ring or early trophozoite stages and to ensure DNA replication for the production of daughter progeny had not yet occurred. Two mice from these treatment groups died before the end of this experimental period: Mouse 17 died at 10 days PI; Mouse 18 died at 12 days PI. Both mice were infected with genotype CR.

Polymerase chain reaction assays (Drew and Reece 2007) were used to distinguish and quantify asexual stages and gametocytes produced by each clone throughout infection. Total RBC densities were estimated using flow cytometry Coulter Counter, Beckman Coulter [see (Ferguson et al. 2003)] and densities of immature RBCs (reticulocytes) were estimated from Giemsa stained thin blood smears. Reticulocytes are distinguished from normocytes based on their morphology; normocytes are small and stain grey, where as reticulocytes are bigger, stain blue and often still contain the remnants of nuclear material. All procedures were conducted in accordance with the United Kingdom Animals (Scientific Procedures) Act 1986.

### Statistical models

We modelled gametocyte density (*G*) (parasites/ml of blood) as time-delayed functions of asexual density (*M*) and RBC (*X*). The delay is the interval between commitment to gametocytogenesis and the appearance of mature gametes in the circulation. We modelled gametocyte density *G* at day *t* in mouse *i* as a function of the asexual parasite density (*M*) *j* days previously (eqn 1) or a combination of *M* and *X* (eqn 2);


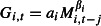
(1)

or


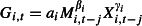
(2)

where *X* was total RBC (*T*), reticulocytes (*R*), or mature RBC (normocytes, *N*). To normalize residuals, we log-transformed the observables:


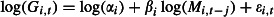
(3)


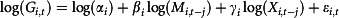
(4)

The residuals, ε_*i,t*_ were assumed independent and normally distributed with mean zero and unknown variance, and the parameters α, β and γ were modelled to include mouse (*i*) as a normally distributed random effect.

Density dependence was modelled with the exponents β and γ. If 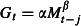, the *per capita* rate of conversion (the proportional investment in gametocytogenesis) at day *t − j* is proportional to 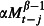. Thus, β = 1 in eqn 1 implies that a constant proportion of merozoites convert to gametocytes during each replicative cycle with no adjustment in response to total parasite density. β > 1 implies a positive correlation between the proportional investment and total parasite density, and β < 1 a negative correlation. In the two-factor models, the quantity *X*^γ^ influences proportional investment multiplicatively. A value γ < 0 means the parasite increases its proportional investment in gametocytes when RBC resources decline. The constant of proportionality α includes the mortality of gametocytes between the initiation of their development and their observation.

### Parameter estimation

Parameters were estimated using a linear mixed effects approach. Zero values were assumed to be 0.5 of the smallest observed value of that covariate in the entire data set. Model selection (using the Bayesian Information Criterion (BIC), with differences of 3 or greater considered significant) was robust to changes in this definition of the limit of detection. Analyses were performed in R (The R foundation for statistical computing; http://www.R-project.org) using the *lmer* package.

## Results

**Figure 3:**
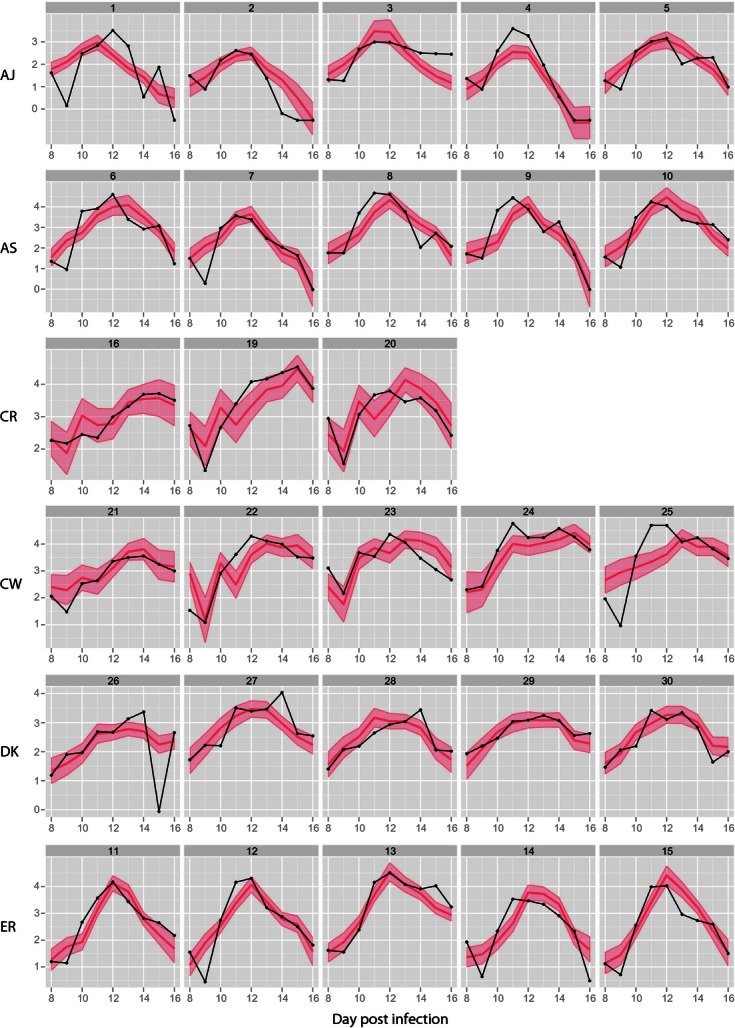
Best-fits to gametocyte density for each mouse, by strain. *Y* axes show the logarithm to base 10 of gametocyte densities per ml of blood. Dark red lines are best-fit predictions and shaded regions are 95% uncertainty envelopes obtained by resampling the parameters from their empirical distributions.

Parameters were estimated for each genotype separately, because it has been demonstrated previously that they differ in their patterns of gametocytogenesis (Pollitt et al. 2011b). Time lags of 2 or 3 days provided the best description of the data, with lags of 1 or 4 days yielding consistently poorer fits. Data were available from day 5 to 16 PI, and so to allow comparison of models with different time lags, all estimates were obtained using the gametocyte densities between days 8 and 16 PI. For all strains, the best-fitting models were of the form of eqn 2, with the covariate *X* being either total RBC or reticulocytes, and with time lags of 2 or 3 days (Table 1 and Fig. 3). Analysis of different error structures indicated that only the intercept log(α) was required as a random effect.

**Table 1:** Summary of models of gametocytogenesis. The best-fitting models for all strains were of the form 
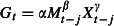
 (eqn 2), where *X* was total red blood cells (RBC) or reticulocytes

Strain	Resource sensitivity	Time lag, h	Exponent β (influence of parasite density)	Exponent γ (influence of resource)
AJ	Total RBC	48	0.61 (0.08)	−3.14 (0.58)
AS	Total RBC	72	0.94 (0.14)	−3.40 (0.53)
CR	Reticulocytes	48	0.76 (0.20)	1.70 (0.23)
CW	Reticulocytes	48	0.49 (0.17)	1.60 (0.22)
DK	Total RBC	72	0.92 (0.17)	−2.30 (0.50)
ER	Total RBC	72	0.50 (0.13)	−4.50 (0.43)

Standard errors on parameter estimates are shown in parentheses.

### Dependence of proportional investment on parasite density

AS and DK had fitted exponents *β* close to unity (Table 1), suggesting no direct effect of parasite density on gametocyte investment in these genotypes. For the remaining genotypes 0.5 < β < 1, showing that proportional investment were inversely but weakly related to parasite density; when RBC numbers are controlled for, we find no evidence for increased investment in transmission when parasites are abundant, and find that in most genotypes reproductive restraint is exercised when parasites are abundant.

### Dependence of proportional investment on red blood cell counts

Four genotypes (AJ, AS, ER and DK) exhibited a strong inverse dependence of proportional investment on total RBC numbers, delayed by 72 h (AS, DK and ER) or 48 h (AJ). Thus, as the host becomes more anaemic, the more these genotypes invest in transmission. CR and CW, however, showed a positive dependence of proportional investment on reticulocyte densities 48 h previously (Table 1).

### Correlations between covariates

We found negative correlations between the logarithms of asexual parasite density (*M*) and total RBC in AJ, AS, CW and ER (*P* < 0.01) and between asexual parasites and reticulocytes in all genotypes (*P* < 0.001). To investigate the robustness of the dependencies established in Table 1, first we confirmed that for all genotypes, when comparing the estimate of the parameter β in the simpler model of eqn 1 to that in eqn 2 for each value of the time lag *j*, the direction of the dependence was unchanged. That is, the 95% confidence intervals for β included unity for AS and DK, and lay entirely below unity for AJ, CR, CW, ER. Similarly, we estimated the parameter *γ* in models of the form 
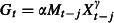
 – that is, forcing *β* = 1 and modelling conversion as a function of *X* alone. Again, the dependencies remained – that is, a strong negative correlation with total RBC at both 48 and 72 h time lags for AJ, AS, DK, ER (γ < 0 for all strains, *P* < 0.001) and a significant positive correlation with reticulocytes 48 h previously for CR and CW (γ > 0, *P* < 0.001 for both strains).

### Model validation and robustness: testing the assumption of a constant probability of loss between commitment to gametocytogenesis and maturation

**Figure 4:**
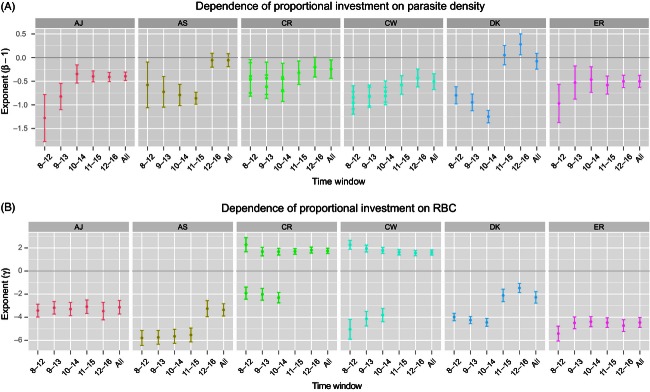
Best-fit exponents β and γ in the model 
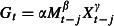
, stratified by 5-day time windows. For the first three time windows (days 8–12, 9–13, 10–14), the CR and CW data were described by two models with equivalent support, as described in the text. Upper panel – the dependence on the proportional investment in gametocytes on the asexual parasite density, *M*. Gametocyte numbers are modelled as *M*^β^ and so the probability per asexual parasite of committing to gametocytogenesis varies as *M*^β−1^. A negative value for the exponent β *−* 1 implies an inverse relation between parasite density and transmission investment. Lower panel; the per-parasite probability of becoming a gametocyte is modelled as proportional to *X*^γ^ where *X* is either total red blood cells (RBC), mature RBC or reticulocytes. Negative values of γ were obtained for genotypes AJ, AS, DK and ER across the whole time course, showing that these genotypes invest more in transmission as RBC resources decline. For genotypes CR and CW, positive values of γ were associated with reticulocytes; negative for total RBC.

We used a single model and parameter set to describe gametocytogenesis over days 5–16 of infection, and assumed that a constant proportion of the parasites that commit to gametocytogenesis at day *t − j* survive to be observed at day *t*. This mortality rate is contained in the parameter α (eqns 1 and 2). However, the analysis may have been confounded by changes in α over time. For example, specific antibody responses develop slowly during the first 2 weeks of infection and may drive a progressive increase in the *per capita* rate of removal of gametocytes. We wanted to validate the assumption that, whatever changes in mortality may occur during infection, the same dependence of gametocytogenesis on the environmental parameters holds. To do this, for each strain we fitted models to 5-day windows of data, first modelling gametocyte density on days 8–12 as functions of covariates on days 5–9, then on days 9–13 as functions of the covariates on days 6–10, and so on up to days 12–16 inclusive. We then compared the estimates of the exponents β and λ obtained from these restricted time series with the estimates obtained from the entire data set (Fig. 4).

The analysis showed that (i) AJ and ER were consistently best described by their globally best-fitting models, with a relatively weak negative dependence of proportional investment on parasite density (Fig. 4A) and strongly negative dependence on total RBC (Fig. 4B); (ii) At later timepoints, CR and CW were described equivalently by a positive dependence of proportional investment on reticulocytes lagged by 48 h or a negative dependence on total RBC lagged by 72 h, and by their global best-fitting model (positive dependence on reticulocytes lagged by 48 h) thereafter (Fig. 4B); (iii) until the last window of days 12–16, AS and DK were both described best by a 48 h lagged RBC count with β < 1, rather than the global best-fitting model of 72 h-lagged total RBC count with *β* = 1 (Fig. 4B); and (iv) the parameter α declined with time for all strains except CR (approximate fractional decrease in α between intervals day 8–12 and day 12–16; AJ, 25%; AS, 20%; CW, 10%; DK, 8%; ER, 10%). This decline in α suggests that either proportional investment declines with time, independent of the covariates, or that gametocyte mortality increases during infection. As gametocyte-specific immune responses are developing during the first 2 weeks of infection we favour the latter interpretation.

We can draw four conclusions here. First, irrespective of the time delay, for four strains we make the robust conclusion that throughout the observed course of infections, proportional investment in reproduction increases as RBC resources decline. Second, the variability in the time lag (48 or 72 h) suggests one or more of the following are at play; variation in maturation time, gametocytes may survive in the circulation for longer than 24 h, merozoites may precommit their progeny to gametocytogenesis, or the assays employed detect gametocytes at slightly different maturation stages in different genotypes. Expression and morphology data demonstrate that transcription of assayed genes occurs before gametocytes reach maturity, but the precise timing and whether there is genetic variation in the timing are yet to be determined. Third, the variability in the exponent β suggests that the negative dependence of reproductive investment on parasite density is weak or may be confounded by other factors such as immune responses. Finally, over the course of infections with the two strains CR and CW, predictors of gametocytes moved between either a positive dependence on reticulocytes lagged 48 h or negative dependence on total RBC lagged 72 h. Consistent with these results, for these two strains only there were weak but significant negative correlations between reticulocytes and total RBC 1 day earlier (data not shown). Further, restricting the CR and CW analyses to days 5–13 yielded an overall negative dependence on total RBC, as for the other strains [delay of 72 h; *γ* = −2.0 (CR) and *γ* = −4.1 (CW)].

### Relation to previous studies

In a previously published analysis of the same data set (the main purpose of which was to look at the impact of competition on investment in gametocytes), proportional investment was found to correlate positively with the availability of total RBC and the proportion of RBC that are reticulocytes for five of the six genotypes when in single infections (Pollitt et al. 2011b). The discrepancy between that study and this one may arise from (i) the use of the formalism described in Buckling et al. (1999b), which assumes a constant linear relation between asexual numbers 2 days apart – this corresponds to geometric growth (that is, RBC are assumed to never be limiting); and (ii) the use of a linear model to assess the effect of RBC on gametocyte investment. In contrast, our method requires no assumptions regarding asexual dynamics, and allows for nonlinearities in the dependence of proportional investment in gametocytes on parasite density and host factors, via the exponents β and γ. There is no reason to assume that proportional investment are linearly related to RBC numbers, and indeed we identified strong nonlinear dependencies.

## Discussion

Predicting when and how a sexually reproducing parasite's transmission investment strategy changes is important because this trait underpins disease severity, epidemiology, and may help parasites evade control measures. Understanding parasites' responses to environmental cues can therefore inform the design of vaccines and drug treatments. Here we have addressed this problem in a rodent malaria model, using a novel method of analysis of transmission strategies that can be applied to other host-parasite systems. Our analysis of infections with six genotypes of *P. chabaudi* suggests that both RBC availability and asexual density influence the parasite's allocation of resources into reproduction. We find that investment in transmission increases rapidly as RBC numbers decline, and for some genotypes increases, albeit more weakly, at low parasite densities. We validated the models by showing that these relationships also hold within shorter time windows between days 5–16 PI. We also found evidence for gametocyte mortality increasing with time, likely due to developing immune responses.

Are these patterns of investment adaptive for the parasite? The default evolutionary explanation for plasticity in gametocyte investment is that parasites demonstrate ‘terminal investment’ in response to an emergency situation that threatens its survival within the host. Indeed theory predicts that parasites should increase investment when the parasite approaches lethal density (here, by a combination of anaemia and/or immunopathology) or shortly before it is cleared by an immune response (Koella and Antia 1995). At first sight our results seem to support this hypothesis – investment in transmission increases as RBC resources and/or parasite numbers decline, both possible signifiers of a decrease in habitat quality. However, this may not be the case, for the following reasons. First, we may be describing causal relationships using surrogates of environmental variables that are the true determinants of parasite behaviour. For example, the development of host anaemia may correlate with increasing concentrations of transmission-blocking immune factors, and so increased investment in gametocytes may then be a response to maintain the probability of successful infection of the mosquito vector (Ramiro et al. 2011). Another alternative is that the development of host anaemia signifies the imminent appearance of reticulocytes. If the parasite can utilize this type of RBC, and in particular if reticulocytes support gametocyte development better than normocytes [as is the case for, e.g. *Plasmodium vivax* (Bousema and Drakeley 2011) and clones of *P. falciparum* (Trager and Gill 1992)], increased investment represents strategic use of resources. This may also explain the positive correlation between proportional investment and reticulocytes for CR and CW. Second, we found that asexual density was either a weak negative influence on gametocyte investment (strains AJ, CR, CW, ER) or had no significant effect (AS, DK). Given that most malaria infections are not lethal, we expect selection for terminal investment would be driven by the threat of imminent clearance from the host, resulting in gametocyte investment showing a stronger dependence on asexual densities than on environmental variables. Third, under more stressful in-host conditions than those reported here (competition in co-infections) some of the genotypes studied here prioritize their in-host survival over short-term transmission (Reece et al. 2010; Pollitt et al. 2011a,b). Therefore, we suggest that empirical work is now needed to examine parasite responses to perturbations in immune factors and preferred RBC ages.

### The timing of developmental cues

We found comparable support for models that explained gametocyte densities as functions of the asexual densities and host environment 48 and 72 h previously. Current understanding of the *P. chabaudi* life cycle is that gametocytes take 48 h to develop following infection of a red blood cell. However, the period during which *Plasmodium* commits to the sexual stage, or how this decision can be influenced, is unclear. All *P. falciparum* parasites within one infected cell result from a single developmental choice, suggesting that initiation of differentiation happens before replication within a red blood cell (Silvestrini et al. 2000; Smith et al. 2000). Further, it has been suggested that merozoites may be developmentally precommitted to differentiation, imprinted with cues received by the parental parasites (Dixon et al. 2008), allowing for the possibility of a 72 h delay between triggering of differentiation and the appearance of mature gametocytes. Alternatively, if mature gametocytes remain in circulation sufficiently long, circulating gametocyte densities may be superpositions of two cohorts that committed 2 and 3 days previously. However, despite the variation in the best-fitting time lag across strains, we found consistent nonpositive dependencies of proportional investment in transmission on RBC and asexual parasite densities.

### Gametocytes as a proxy for transmission; from rodent to human malarias

While gametocytes are necessary for transmission, there are some issues with their use as a proxy for transmission. Gametocyte density is positively correlated with infectivity to mosquitoes in this *P. chabaudi* model (Taylor et al. 1997; Mackinnon and Read 1999) and in *Plasmodium mexicanum* infections of lizards (Schall 2000). However, the data are more equivocal in human malaria species. A log-sigmoid relationship between gametocyte density in blood and infectivity was observed in field studies of *P. falciparum* in humans (Jeffery and Eyles 1955), but there was also an independent effect of duration of infection on infectivity; fever, but not gametocyte count, was observed to influence the infectivity of *P. vivax* in humans (Collins et al. 2004); and in *P. falciparum* infections, the variance and not the mean gametocyte density was found to account for most variation in infectivity between groups of individuals (Paul et al. 2007). Host anaemia can also influence *P. falciparum* transmission, independently of gametocyte density (Drakeley et al. 1999).

The effects of immunity to different developmental stages of gametocytes may underlie these observations (Piper et al. 1999; Drakeley et al. 2006). Measurements of transmission at times following drug treatment have shown that a *P. falciparum* gametocyte's reproductive potential varies with its maturity (Hallett et al. 2006). Immune effector molecules in serum such as reactive nitrogen species and TNF-α also influence gametocyte fertility in *Plasmodium berghei* infections of rodents (Ramiro et al. 2011). Further, the sex ratio of gametocytes is important for transmission, because the density of females places an upper limit on the intensity of mosquito infection (Reece et al. 2008; Mitri et al. 2009). Relative investment in males and females varies with infection genetic diversity, host anaemia and is also likely to be shaped by immune responses (Gardner et al. 2003; Paul et al. 2003). Therefore, individuals with differing degrees of anaemia, coinfection and immunity to gametocytes may have variable infectivities after total gametocyte numbers are controlled for. The precise role of immunity in gametocyte investment remains to be established, but an understanding of the dynamics of parasites and the immune responses to them is likely essential for identifying and explaining transmission strategies in chronic malaria infections (Paul et al. 2007; Bousema and Drakeley 2011). However, in our system, we are dealing with acute infections of isogenic animals, in which high-affinity antibody responses have yet to fully develop. Therefore, we expect gametocyte density to be the key determinant of transmission success in this phase.

### Virulence correlates with the degree of influence of asexual parasite density

The six genotypes used here vary in their level of virulence, from weakly (AS, DK, CR) to moderately (CW) to highly virulent (AJ, ER) (Mackinnon and Read 2003; Mackinnon et al. 2005; Bell et al. 2006). Given the trade-off parasites are expected to experience between transmission and virulence (Bull 1994; Day 2003; Alizon and van Baalen 2008) it may be thought that genotypes exhibiting similar levels of virulence will modify their investment in gametocytes in response to environmental cues in the same manner. Indeed we found the strength of the dependence of the rate of gametocytogenesis on parasite density is associated with virulence. The two least virulent strains (AS and DK) show the lowest sensitivity of reproduction to asexual density (*β −* 1 closest to zero), and the most virulent genotypes (AJ, CW, ER) show the highest (*β −* 1 most negative). In contrast, sensitivity to RBC or reticulocyte availability did not correlate with virulence. The harm these strains do to their hosts is positively correlated with their competitive ability in mixed infections (Bell et al. 2006), and so it is plausible that virulent strains can ‘afford’ increased investment in transmission when pathogen densities are low. The genetic variation in these reaction norms suggests that genotypes may divergently evolve and have the potential to respond to selection pressures.

### Top-down versus bottom-up control

Pressures on parasites within hosts are often partitioned conceptually into top-down (immune-mediated) or bottom-up (resource limitation) forces (Haydon et al. 2003; Graham 2008). Without readouts of immune responses, we cannot discount the possibility that the RBC covariates in our models are also correlates of immune responses, as discussed previously. The distinction between the immune-and resource-mediated pressures may also be a blurry one, given that excessive immune responses, in particular relatively indiscriminate responses by splenic macrophages, may give rise to extensive lysis of uninfected RBC and loss of parasite resources (Schofield and Grau 2005; Evans et al. 2006). Thus, early in the infection, RBC loss and immune responses may be positively correlated. Indeed immune-mediated destruction of uninfected susceptible cells may be a host strategy to create a fire-break that limits the spread of intracellular pathogens (Handel et al. 2009). As anaemia worsens, the parasite may also have to invest more in overcoming transmission-blocking immune factors, and both innate and adaptive immune responses to the parasite will limit transmission (Carter et al. 1979).

## Summary

Further experiments are required to fully disentangle the complex mechanisms behind, and fitness consequences of, plasticity in the transmission strategies of *P. chabaudi*. Future work should focus on the effects of parasite age preference for RBC and the role of host immunity in determining transmission effort, and especially how these variables shape the intensity and prevalence of infections in mosquito vectors. Experiments manipulating RBC densities (for example with Erythropoietin or phenylhydrazine), and manipulations of host immunity (through vaccination, drugs and infective doses) will undoubtedly prove important next steps in furthering our understanding of the ecological and evolutionary processes underlying parasite transmission strategies.
